# Inverse Mixed-Solvent Molecular Dynamics for Visualization of the Residue Interaction Profile of Molecular Probes

**DOI:** 10.3390/ijms23094749

**Published:** 2022-04-26

**Authors:** Keisuke Yanagisawa, Ryunosuke Yoshino, Genki Kudo, Takatsugu Hirokawa

**Affiliations:** 1 Department of Computer Science, School of Computing, Tokyo Institute of Technology, 2-12-1 Ookayama, Meguro-ku 152-8550, Tokyo, Japan; yanagisawa@c.titech.ac.jp; 2 Faculty of Medicine, University of Tsukuba, 1-1-1 Tennodai, Tsukuba 305-8575, Ibaraki, Japan; yoshino.r.aa@md.tsukuba.ac.jp; 3 Transborder Medical Research Center, University of Tsukuba, 1-1-1 Tennodai, Tsukuba 305-8577, Ibaraki, Japan; 4 Physics Department, Graduate School of Pure and Applied Sciences, University of Tsukuba, 1-1-1 Tennodai, Tsukuba 305-8571, Ibaraki, Japan; s2120165@s.tsukuba.ac.jp

**Keywords:** mixed-solvent molecular dynamics, interaction pattern analysis, residue interaction profile, visualization

## Abstract

To ensure efficiency in discovery and development, the application of computational technology is essential. Although virtual screening techniques are widely applied in the early stages of drug discovery research, the computational methods used in lead optimization to improve activity and reduce the toxicity of compounds are still evolving. In this study, we propose a method to construct the residue interaction profile of the chemical structure used in the lead optimization by performing “inverse” mixed-solvent molecular dynamics (MSMD) simulation. Contrary to constructing a protein-based, atom interaction profile, we constructed a probe-based, protein residue interaction profile using MSMD trajectories. It provides us the profile of the preferred protein environments of probes without co-crystallized structures. We assessed the method using three probes: benzamidine, catechol, and benzene. As a result, the residue interaction profile of each probe obtained by MSMD was a reasonable physicochemical description of the general non-covalent interaction. Moreover, comparison with the X-ray structure containing each probe as a ligand shows that the map of the interaction profile matches the arrangement of amino acid residues in the X-ray structure.

## 1. Introduction

Drug development is a cost-intensive and time-consuming process. The cost is estimated to be more than USD two billion, and it may take 10–15 years for a new drug to reach the market [1]. To reduce costs, computational techniques have been applied in various drug development studies, and many studies have successfully discovered new therapeutic compounds using these techniques [2,3,4,5,6,7,8,9,10]. In the early stages of drug discovery, virtual screening (VS) techniques such as docking simulations are frequently used to discover seed compounds [11,12,13]. Kelly et al. showed that high-throughput virtual screening (HTVS) produced more than 10-fold hit rates compared to traditional HTS [14]. Computational methods for lead optimization to improve the activity of compounds are also proposed, such as MP-CAFEE [15], free energy perturbation (FEP) [16,17], and quantum mechanical (QM) methods [18]. These methods focused on the binding affinity estimation of given candidate compounds with considerable computational cost; however, methods guiding or proposing the next substituent of hit compounds in the lead optimization phase are still evolving.

To improve activity and selectivity, structural optimization that is strongly and specifically directed at the target protein is necessary. Therefore, the mechanism by which proteins recognize compounds and the prediction of protein–ligand binding modes are important in lead optimization. Several studies have been reported that comprehensively analyze interaction patterns from protein–ligand complex structures registered in the Protein Data Bank (PDB) [19]. Imai et al. focused on 14 polar and aromatic amino acid side chains and carried out contact analysis for protein–ligand complex crystal structures in the PDB [20]. Wang et al. generated 4032 fragments from 71,798 ligands and obtained fragment–residue interaction profiles [21]. Furthermore, Kasahara et al. reported that 63.4% of the ligand atoms exhibited one or more interaction patterns, 25.7% of the ligand atoms interacted without patterns, and the rest had no direct interaction [22]. However, these interaction patterns have been analyzed only from known databases, and the analysis is limited to the well-experimented functional groups. In addition, the interaction patterns of the functional groups investigated in these studies were studied while binding to the target as part of the compound, and the environment of the protein to which functional group fragments match has not been directly investigated.

Mixed-solvent molecular dynamics (MSMD) simulations involve MD in the presence of explicit water molecules mixed with probe molecules or functional group fragments such as for hotspot detection [23,24] and binding site identification [25,26]. MSMD considers the flexibility of proteins and can discover hotspots where probes can bind. These hotspots indicate the protein environment preferred by specific probes. Thus, by analyzing the interaction pattern between the probe and the protein environment sampled by MSMD, it is possible to analyze the binding ability and interaction pattern of individual probes, not part of the functional group of compounds. In particular, by applying drug-like probes with few reported crystal structures to MSMD, the environment of the protein binding site to which the unique probe binds can be sampled.

In this article, we propose an “inverse” MSMD for analyzing a probe’s preference of interaction patterns. First, MSMD simulations with 15 diverse proteins were performed to sample various protein residue environments preferred by the probe. The residue environments were then integrated to a residue interaction profile, followed by the visualization of it. We assessed the proposed analysis using three probes with an aromatic ring: benzamidine, catechol, and benzene. Their residue interaction profiles provided a physicochemical account of general non-covalent interactions, such as electrostatic interactions, hydrogen bonding interactions, and amide-π stacking interactions. Moreover, the profiles were consistent with the experimental co-crystalized structures, which supports the ability of the proposed method to detect the actual interaction patterns of functional groups. This is the first proposal and demonstration of the use of inverse MSMD.

## 2. Materials and Methods

### 2.1. Preparation of Proteins

The selection of proteins used in MSMD sampling is crucial to obtain the various residue environments utilized to construct a residue interaction profile. We chose 15 proteins that were previously selected by Soga et al. [27] because they collected the proteins considering their diversity. A list of proteins is shown in Table 1. All proteins were pre-processed using the following procedure: Protein Preparation Wizard and Prime [28] in Schrodinger suite 2020-3 (Schrodinger, Inc., New York, NY, USA) were used to fill the missing loops, side chains, and atoms for all of the selected proteins. N- and C-termini were capped using N-methyl amide (NME) and acetyl (ACE) capping groups, respectively. Subsequently, the ligands, co-factors, and additive molecules were removed. Hydrogens were placed with consideration of hydrogen bonding and ionization states of pH = 7 with PROPKA [29]. Water molecules with less than two hydrogen bonds to a protein in the crystal structures were removed, followed by structure optimization with the OPLS3e force field. Note that the N-terminal of glycoside hydrolase (PDBID: 1H4G) and C-terminal of endo-1,4-β-xylanase A precursor (PDBID: 1E0X) were removed before the procedure because of non-standard amino acid and missing main chain atoms, respectively.

### 2.2. Preparation of Probes

The probe of interest was then pre-processed. The restrained electrostatic potential (RESP) procedure in the Antechamber module of AmberTools18 [30] was employed to fit the partial charges to the electrostatic potential, which was calculated using Gaussian 16 Rev B.01 [31]. First, all probe structures were optimized at the B3LYP/6-31G(d) level. Then, the electrostatic potentials were calculated at the HF level using the optimized structures. The centers of the electrostatic potentials were placed at the center of each atom. Additional force field parameters for the probes were derived using the general AMBER force field 2 (GAFF2), unless otherwise stated.

### 2.3. Mixed-Solvent Molecular Dynamics (MSMD)

We conducted MSMD using the protocol of EXPRORER [32]. It is worth noting that the initial positions of the probes affect the results, especially in short MD simulations, and this initial position dependency influences the convergence of the results of the analysis. To achieve efficient sampling, the following protocol was independently performed 20 times with different initial probe coordinates. The procedure was divided into three steps, as described below.

#### 2.3.1. Initial System Generation

The probes were randomly placed around the protein at a concentration of 0.25 M using PACKMOL 18.169 [33]. The high concentration enables effective sampling of residue environments. Second, the system was solvated with water using the LEaP module of AmberTools18. The Amber ff14SB force field and the TIP3P model [34] were used for the protein and water molecules, respectively. Additionally, a Lennard–Jones force field term with the parameters (ϵ = 10^−6^ kcal/mol; R_min_ = 20 Å) was introduced only between the center of the probes to prevent their aggregation.

#### 2.3.2. Minimization, Heating, and Equilibration

After the construction of the initial structures, the systems were minimized to include 200 steps using the steepest descent algorithm with harmonic position restraints on the heavy solute atoms (force constant, 10 kcal/mol/Å^2^), and then the systems were minimized a further 200 steps using the steepest descent algorithm without any position restraints. After minimization, the system was heated gradually to 300 K during 200 ps constant-*NVT* MD simulations with harmonic position restraints on the solute heavy atoms (force constant, 10 kcal/mol/Å^2^). During the subsequent 800 ps constant-*NPT* MD simulations at 300 K and 10^5^ Pa, the force constants of the position restraints were gradually reduced to 0 kcal/mol/Å^2^. The P-LINCS algorithm [35] was used to constrain all bond lengths involving hydrogen atoms, which allowed the use of 2 fs time steps. Temperature and pressure were controlled using a stochastic velocity-rescaling (V-rescale) algorithm [36,37,38] and a Berendsen barostat [39], respectively. Simulations were performed using GROMACS 2019.4 [40]. The ParmEd module [41] was used to convert the AMBER parameter/topology file format to that used by GROMACS.

#### 2.3.3. Production Run

After equilibration, 40 ns constant-*NPT* MD simulations at 300 K and 10^5^ Pa without position restraints were performed. All settings were the same as the initial equilibration step, but a Parrinello–Rahman barostat [42] was used instead of a Berendsen barostat. Snapshots were taken every 10 ps in the 20–40 ns; thus, 2000 snapshots were produced per MSMD simulation.

### 2.4. Inverse MSMD: Construction of Residue Interaction Profile

**Figure 1:**
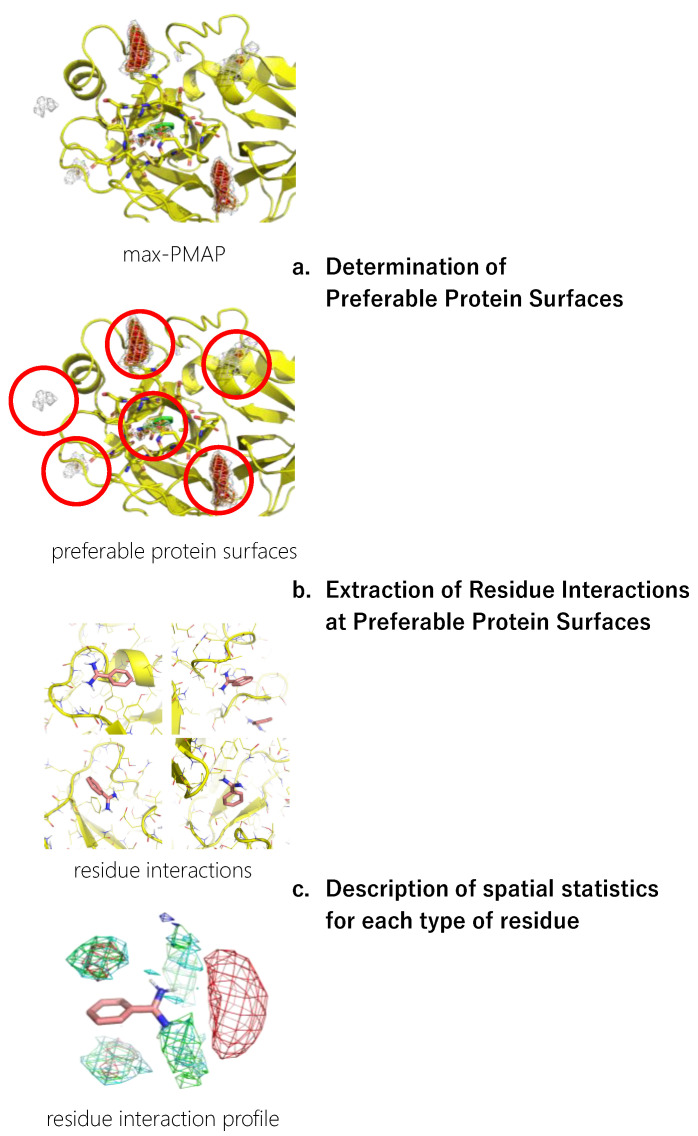
A workflow of inverse MSMD to construct a residue interaction profile.

The workflow for constructing a residue interaction profile from the MSMD simulation is shown in Figure 1. The detailed procedure is described in this section.

#### 2.4.1. Determination of Preferable Protein Surfaces

The concentration of probes in the MSMD simulation was unrealistically high, which could cause artificial interaction profiles even though the concentration is sometimes used in other MSMD protocols [43]. To omit such artifacts, we limited the residue environment sampling based on the binding preference of the probes. First, the spatial probability distribution map (PMAP) was calculated using the following procedure: Atoms in the snapshots were binned into 1 Å × 1 Å × 1 Å grid voxels, and the voxel occupancy of probe heavy atoms was calculated. To focus on the protein surface, V was a set of voxels within 5 Å from the protein atoms, and the values at voxel v∉V were discarded. Then, the values were scaled such that the summation of voxels in V was 1.0.

**Figure 2:**
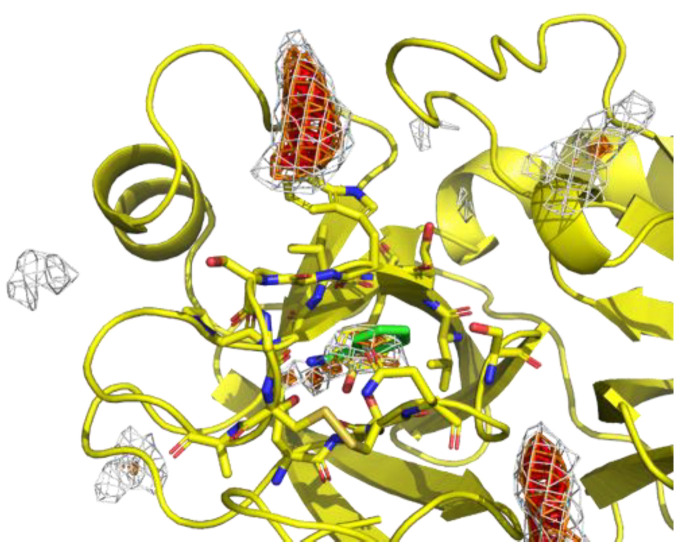
An example of preferable protein surfaces based-on max-PMAP. Positions shown as meshes are preferable protein surfaces, where the value of max-PMAP is higher than a threshold. The gray, orange, and red meshes show the same max-PMAP but different thresholds.

Probes were placed uniformly among the system, resulting in the underestimation of the probability at deep pockets where access is difficult. Thus, to reduce the underestimation, the largest value among the 20 PMAPs generated from each independent trajectory was stored for each voxel in V in the second step. The product was named max-PMAP. Even for a deep pocket where a probe will bind strongly but will rarely approach, a considerable value of voxel v of max-PMAP was observed if the binding occurred only at least once. Note that the summation of voxels V of max-PMAP was greater than 1.0, while it originated from the probability. Finally, we defined a preferable protein surface of a probe as voxels with max-PMAP values equal to or greater than 0.2, which is determined by visual inspection. Note that the preferable protein surface includes surfaces exposed to solvent as well as deeper binding pockets (Figure 2). The regions indicate the protein surfaces where the probe stably exists.

#### 2.4.2. Extraction of Residue Environments at Preferable Protein Surfaces

**Figure 3:**
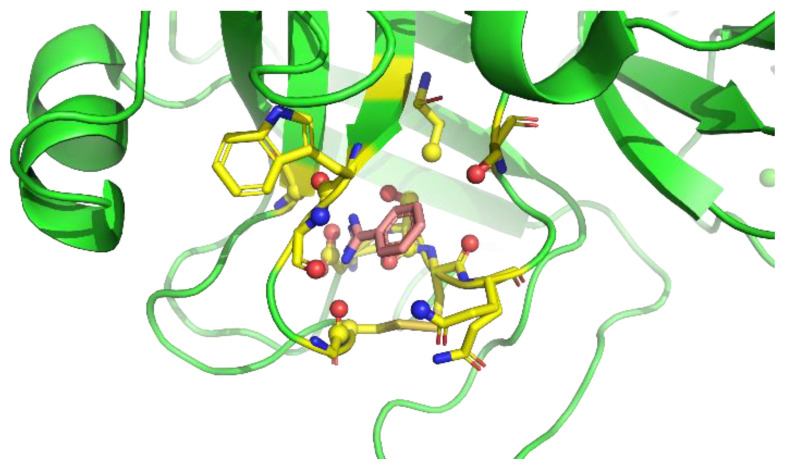
Definition of amino acid residues around the probe. Yellow residues are “amino acid residues around the probe”. Amino acid heavy atoms within 4 Å from any heavy atom of the probe molecule (red molecule) are shown as balls.

Next, the protein residue environments around the probe molecules were extracted. As previously mentioned, we sampled poses on the preferable protein surfaces of the probe. Extraction of preferable protein surfaces of a probe was performed for each snapshot, and the probe and amino acid residues around the probe were extracted after detecting the probe on preferable protein surfaces. Here, we defined “amino acid residues around the probe” as protein residues with at least one heavy atom that is within 4 Å from any heavy atom of the probe molecule (Figure 3).

#### 2.4.3. Description of Spatial Statistics for Each Type of Residue

Finally, a residue interaction profile, or a set of spatial distribution of residues around a probe, was described with the following steps. All the sampled residue environments were aligned by referring to the designated atoms of the probe molecule. The Cβ atoms of each type of residue were spatially binned into 1 Å × 1 Å × 1 Å grid voxels. The types of residues used in this study are listed in Table 2. Note that we used Cβ atoms rather than the side chain atoms (e.g., nitrogen atoms of Lys and aromatic carbon atoms of Phe) because our aim was to analyze the protein environment, and the direction of the tip of the side chains is easily changed.

### 2.5. Implementation

The scripts used to generate a residue interaction profile of a probe were implemented using Python with Biopython [44]. The implementation is included in a GitHub repository EXPRORER_MSMD https://github.com/keisuke-yanagisawa/exprorer_msmd (accessed on 22 April 2022) under the MIT license.

## 3. Results

We tested the proposed method with benzamidine, catechol, and benzene. Ring systems are key scaffold components in medicinal chemistry [45]; therefore, we selected these probes with a ring system as examples of available probes. Note that since the GAFF2 force field incorrectly parameterized the amidino group of benzamidine, we manually assigned “nc” and “cc” GAFF2 atom types for nitrogen and carbon atoms of the amidino group, respectively, to maintain planarity of the functional group. Additionally, structural alignment of the probes was performed with all carbon atoms for benzamidine and with all heavy atoms for catechol and benzene.

### 3.1. Benzamidine: Evaluation of the Method

**Figure 4:**
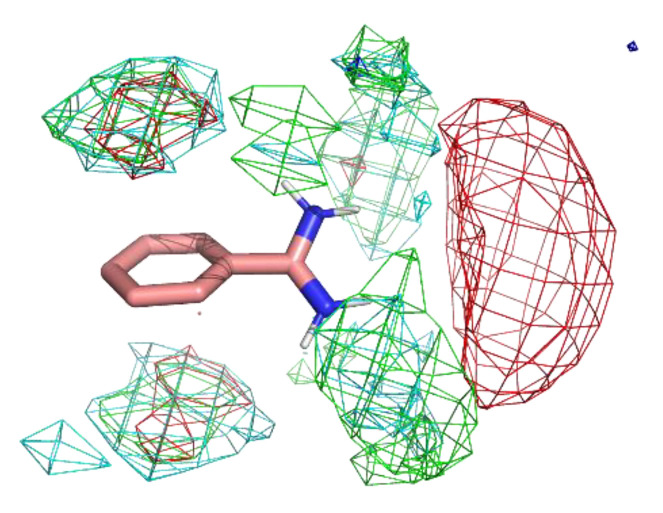
The residue interaction profile of benzamidine. Green, cyan, gray, blue, and red meshes indicate profiles of hydrophobic, hydrophilic, aromatic, basic, and acidic residues, respectively. The molecule structure is that of benzamidine.

**Figure 5:**
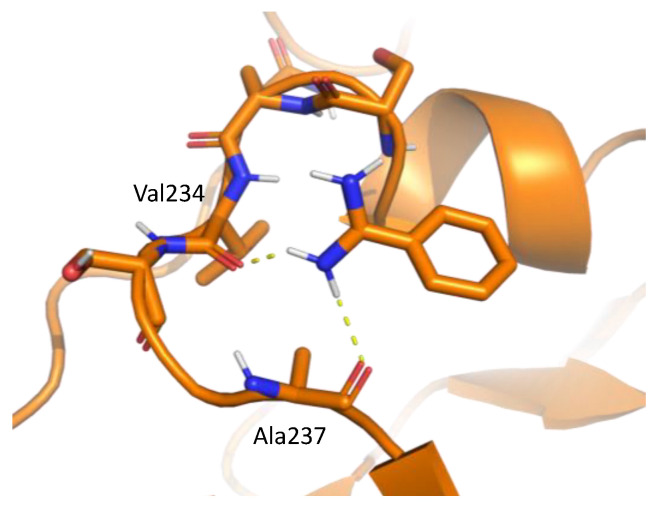
An example of interaction pattern between benzamidine and penicillopepsin (PDBID: 2WEA) obtained from MSMD simulation. Yellow hashed lines show hydrogen bonds.

Figure 4 shows the residue interaction profile of benzamidine. Benzamidine has a basic group and the residue interaction profile correctly and clearly depicted the position of acidic residues. Furthermore, profiles of multiple types of residues were detected among the amidino group, indicating hydrogen bonds between the main chains and the amidino group (Figure 5). For the phenyl group, profiles of acidic, hydrophilic, and hydrophobic residues were detected in the vertical direction of the phenyl group. This suggests that the protocol captured amide-π stacking [46]. Therefore, these profiles can provide a physicochemical account of general non-covalent interactions, and the results demonstrate the validity of the proposed method.

### 3.2. Catechol: Interaction Analysis of Hydroxy Groups

Catechol exists as a substructure in several ligands, such as dopamine. It has two hydroxy groups and a phenyl group. Although it does not have any net charge, which is different from benzamidine, hydroxy groups can form hydrogen bonds, resulting in stable binding to proteins.

**Figure 6:**
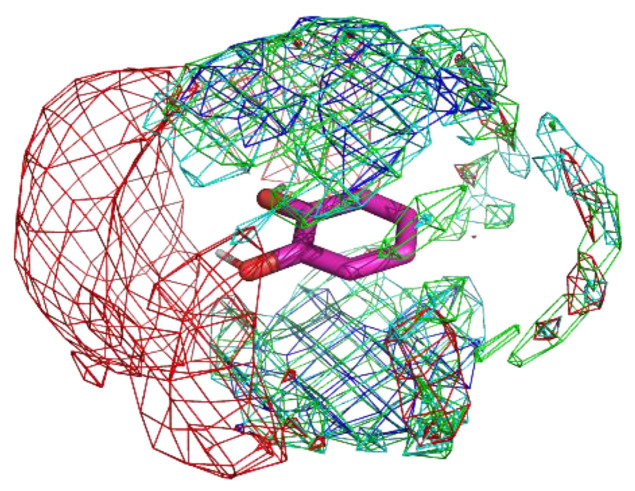
The residue interaction profile of catechol. Green, cyan, gray, blue, and red meshes indicate profiles of hydrophobic, hydrophilic, aromatic, basic, and acidic residues, respectively. The molecule structure is that of catechol.

The residue interaction profile of catechol is shown in Figure 6. Interestingly, the acidic group showed clear localization compared to the other residue groups. This indicates the possibility of detecting not only obvious interactions but also non-intuitive interactions. Further analysis regarding the same is provided in the discussion section. Additionally, the profiles of the phenyl group were similar to those of benzamidine; however, the areas of localization were wider than those of benzamidine. Ghanakota et al. showed that the wider localization of probe atoms can be converted to entropic terms [47]. Thus, the present observations may indicate weaker binding of a catechol substructure to proteins.

### 3.3. Benzene: Interaction Analysis of Phenyl Group Itself

**Figure 7:**
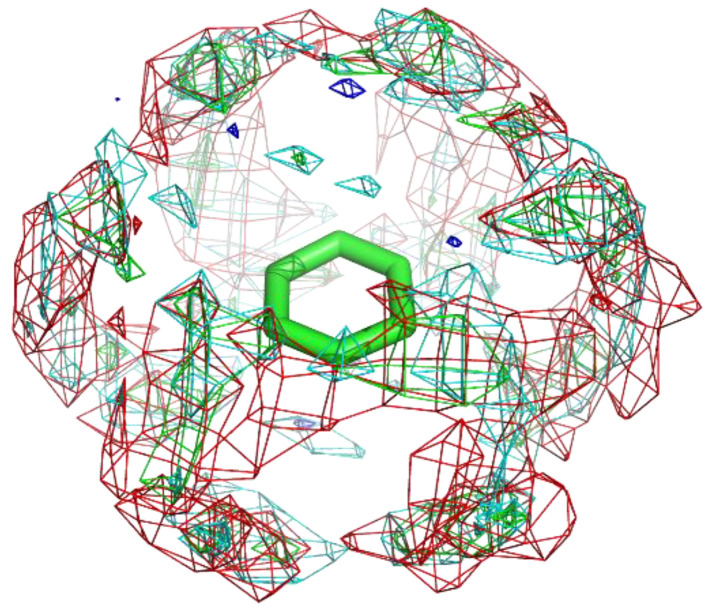
The residue interaction profile of catechol. Green, cyan, gray, blue, and red meshes indicate profiles of hydrophobic, hydrophilic, aromatic, basic, and acidic residues, respectively. The molecule structure is that of catechol.

Benzamidine and catechol both have phenyl groups, and their groups show clear residue interaction profiles. On the other hand, benzene did not display a profile in the vertical direction (Figure 7). Instead, weak profiles were detected in the horizontal direction. This indicates that interaction with only a single amide-π stacking is insufficiently stable at the surface of proteins.

## 4. Discussion

### 4.1. Comparison to Co-Crystallized Structures

We compared the constructed residue interaction profiles and crystal structures for further verification of the appropriateness of the method.

#### 4.1.1. Benzamidine

**Figure 8:**
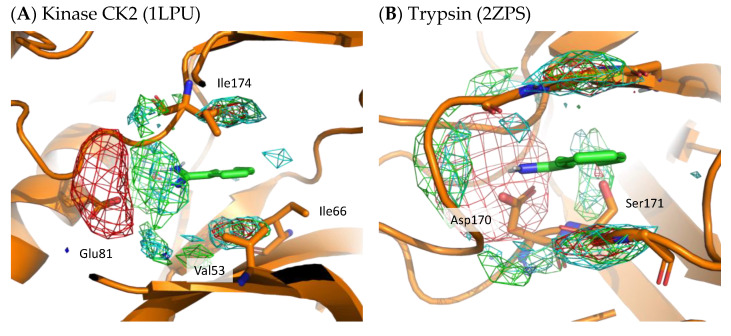
X-ray structure with residue interaction profile of benzamidine obtained from MSMD simulation. (**A**) Kinase CK2 with benzamidine (PDBID: 1LPU); (**B**) trypsin with benzamidine (PDBID: 2ZPS). Green, cyan, gray, blue, and red meshes indicate profiles of hydrophobic, hydrophilic, aromatic, basic, and acidic residues, respectively.

To demonstrate the validity of the residue interaction profile obtained by MSMD, the crystal structures of kinase CK2 (1LPU) and trypsin (2ZPS) with the residue interaction profile are shown in Figure 8. These crystal structures include benzamidine and can be compared to the preferred residue environment obtained by wet experiments. In the residue interaction profile of benzamidine obtained by MSMD, acidic residues are widely present near the amidino group. In kinase CK2 and trypsin, Glu81 and Asp170 are located near the profile of acidic residues. In kinase CK2, Val53, Ile66, and Ile174 are in the profile of hydrophobic residues above and below the aromatic ring of benzamidine. Notably, the profile of hydrophilic residues above and below the aromatic ring was suggested by MSMD. Ser171 of trypsin is located near the aromatic ring of benzamidine, which overlaps with the profile of hydrophilic residues. These two examples of X-ray structures, which are not included in the set of proteins for MSMD simulation, suggest that the residue interaction profile of a probe is reasonable and has generalization performance for any protein.

#### 4.1.2. Catechol

**Figure 9:**
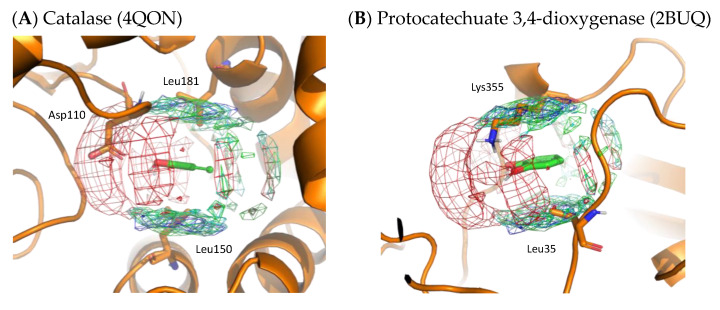
X-ray structures with the residue interaction profile of catechol obtained from MSMD simulation. (**A**) Catalase with catechol (PDBID: 4QON); (**B**) protocatechuate 3,4-dioxygenase with catechol (PDBID: 2BUQ). Green, cyan, gray, blue, and red meshes indicate profiles of hydrophobic, hydrophilic, aromatic, basic, and acidic residues, respectively.

To validate the residue interaction profile of catechol obtained by MSMD, we compared it with X-ray structures that included catechol molecules. Figure 9 shows the X-ray structures of catalase and protocatechuate 3,4-dioxygenase, including catechol. On superimposing the residue interaction profile of catechol over the X-ray structure, Asp110 of catalase is in the profile of acidic residues. The hydrophobic residue interaction profile above and below the aromatic ring contains Leu150 and Leu181 of catalase and Leu35 of protocatechuate 3,4-dioxygenase. In addition, the crystal structure of protocatechuate 3,4-dioxygenase showed that Lys355 overlapped with the interaction profile of the basic residues. The amino acid residues around catechol in these X-ray structures are consistent with the residue interaction profile obtained by MSMD and support the simulation results. Again, these two proteins were not included in the set of proteins for MSMD simulation; thus, it indicates the generalization performance of the profile and the proposed method itself.

### 4.2. Detection of Aromatic Residues’ Profile

Despite several agreements between the X-ray structures and residue interaction profiles obtained from MSMD simulation, the profiles of aromatic residues were not detected sufficiently in spite of the threshold being the same among all residue types. One possible reason is the size of the side chains. Aromatic side chains have a relatively large structure compared to other types of residues, leading to a wider placement of the Cβ atoms. In this study, we aimed to show the residue-based interaction profile; thus, we focused on the Cβ atoms, which are common among almost all residues. However, it is also important to know the interaction profiles of specific atoms of side chains. The implementation already has the functionality to generate the profiles of any specific atoms, as well as Cβ. An exemplary interaction profile of aromatic rings is shown in Figure S1 in Supplementary Materials. Although it is a preliminary visualization and the signal of the profile is not stronger than that of Cβ atoms of acidic residues, the visualization result will suggest the π-π stacking more directly and will be informative for lead optimization.

### 4.3. Consideration of Binding Stability

In this study, we sampled residue environments at preferable protein surfaces to omit artifacts of MSMD simulation. The above results indicate that the sampled residue environments were informative; however, preferable protein surfaces can be classified into two types:

1. Strong binding affinity between a probe molecule and the protein surface, which allows a single probe molecule to bind stably to the surface.
2. Frequent access of probe molecules to the protein surface, which makes multiple probe molecules bind to the protein surface alternatively.

The aim of this sampling was to obtain a residue interaction profile in which a probe molecule binds stably. The protein surfaces of the first type were suitable for this purpose. The surfaces of the second type might be important in providing access to the binding site, but the binding affinity with the probe may not necessarily be strong.

**Figure 10:**
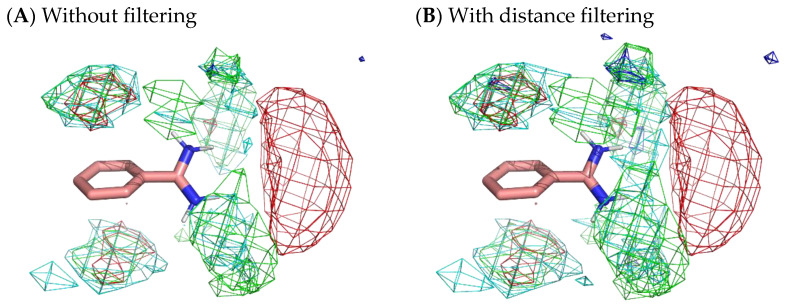
Comparison of residue interaction profiles of benzamidine obtained from MSMD simulation with and without consideration of moving distance of probe molecules. (**A**) Without the selection of stable probe molecules (note that it is the same figure as Figure 4); (**B**) with the selection of stable probe molecules. Green, cyan, gray, blue, and red meshes indicate profiles of hydrophobic, hydrophilic, aromatic, basic, and acidic residues, respectively.

To obtain more appropriate residue interaction profiles, we tried to filter out residue environments whose probes were not stably situated. Here, we defined a stable probe molecule as a probe molecule that moved less than 3 Å from a place where the molecule was 500 ps before. The selection of stable samples omitted 77.4% of the residue environments of benzamidine, 88.0% of those of catechol, and 94.5% of those of benzene. However, Figure 10 reveals only a slight difference with and without filtering. Although filtering slightly enhanced the localization of residues, the results indicated that there was no significant difference between stable surfaces and accessible surfaces.

### 4.4. Substituent Evaluation with Residue Interaction Profiles

As mentioned in the Introduction, structural optimization that is strongly and specifically directed at a target protein is necessary in lead optimization. Residue interaction profiles can help the optimization step by suggesting whether a substituent is feasible for a protein binding pocket space. For instance, superimposing the interaction profile on a co-crystalized structure indicates how well the existing substituent matches the protein surface environment (Figure S2). Furthermore, residue interaction profiles of dozens of probes enable the establishment of a recommendation system by computational substitution of an initial compound and superimposition profiles of corresponding substituents or probes. Therefore, constructing residue interaction profiles of many probes with variation will enhance the impact in lead optimization processes.

## 5. Conclusions

In this article, we proposed inverse MSMD for analyzing a probe’s preference of interaction patterns. Unlike the analysis from known data, such as X-ray, Cryo-EM, and NMR structures, the method can process arbitrary probes, and the results are free from any chemical context of molecules. We assessed the method using benzamidine, catechol, and benzene, resulting in good agreement with the experimental structures. This method indicates where protein surfaces provide suitable binding sites for a probe and this, in turn, can be applied to lead optimization by suggesting substituents based on the vacant spaces of a binding pocket. More precisely, for a target protein surface where the next substituents on hit compounds are placed, the method provides information on which substituents are better in accordance with the residue interaction profiles of multiple probes or possible substituents. The next step in our method will involve proposing a quantitative metric of how well the residue environment and the residue interaction profile match. Furthermore, the construction of the residue interaction profile database is effective for the computational lead optimization process because the profiles can be applied to arbitrary proteins.

## Figures and Tables

**Table 1:** The list of proteins utilized in this and originally collected by Soga et al. [27].

PDB Code	Chain ID	Protein Name
1ZUA	X	Aldo-keto reductase family 1 member B10
1E0X	A	Endo-1,4-β-xylanase A precursor
1BK9		Phospholipase A2, acidic
1TU6	A	Cathepsin K precursor
1W4P	A	Ribonuclease pancreatic precursor
1JZF	A	Azurin precursor
1YMS	A	β-lactamase CTX-M-9a
2WEA		Penicillopepsin
1HEE	A	Carboxypeptidase A1 precursor
1WBI	A	Avidin-related protein 2 precursor
1CXV	A	Collagenase 3 precursor
1H4G	A	Glycoside hydrolase
1TT1	A	Glutamate receptor, ionotropic kainate 2 precursor
2CYB	A	Tyrosyl-tRNA synthetase
1H60	A	Pentaerythritol tetranitrate reductase

**Table 2:** Types of residues used in this study. Note that glycine is not included in this table, since it does not have any Cβ atoms.

Type	Residues
Acidic	Asp, Glu
Basic	Arg, His, Lys
Hydrophilic	Asn, Cys, Gln, Ser, Thr
Hydrophobic	Ala, Ile, Leu, Met, Pro, Val
Aromatic	Phe, Trp, Tyr

## Data Availability

The implementation and experimental data are open-sourced at https://github.com/keisuke-yanagisawa/exprorer_msmd (accessed on 22 April 2022) under the MIT license.

## Supplementary Materials

The following supporting information can be downloaded at https://www.mdpi.com/article/10.3390/ijms23094749/s1.
