# Supplementary material for: Inverse Mixed-Solvent Molecular Dynamics for Visualization of the Residue Interaction Profile of Molecular Probes
Source: Int J Mol Sci. 2022 Apr 26;23(9):4749. doi: 10.3390/ijms23094749 (PMC9103889; doi:10.3390/ijms23094749)
Supplement: Supplementary file 1 [file ijms-23-04749-s001.zip › ijms-1637992-supplementary.pdf]

International Journal of
Molecular Sciences

MDPI

Supplementary Materials

Inverse Mixed-Solvent Molecular Dynamics for Visualization of Residue Interaction Profile of Molecular Probes

Keisuke Yanagisawa [1], Ryunosuke Yoshino [2,3], Genki Kudo [4], Takatsugu Hirokawa [2,3,*]

1 Department of Computer Science, School of Computing, Tokyo Institute of Technology, 2-12-1 Ookayama, Meguro, Tokyo 152-8550, Japan; yanagisawa@c.titech.ac.jp
2 Faculty of Medicine, University of Tsukuba, 1-1-1 Tennodai, Tsukuba, Ibaraki 305-8575, Japan; yoshino.r.aa@md.tsukuba.ac.jp
3 Transborder Medical Research Center, University of Tsukuba, 1-1-1 Tennodai, Tsukuba, Ibaraki 305-8577, Japan
4 Physics Department, Graduate School of Pure and Applied Sciences, University of Tsukuba, 1-1-1 Tennodai, Tsukuba, Ibaraki 305-8571, Japan; s2120165@s.tsukuba.ac.jp
* Correspondence: t-hirokawa@md.tsukuba.ac.jp

Supplementary Figures

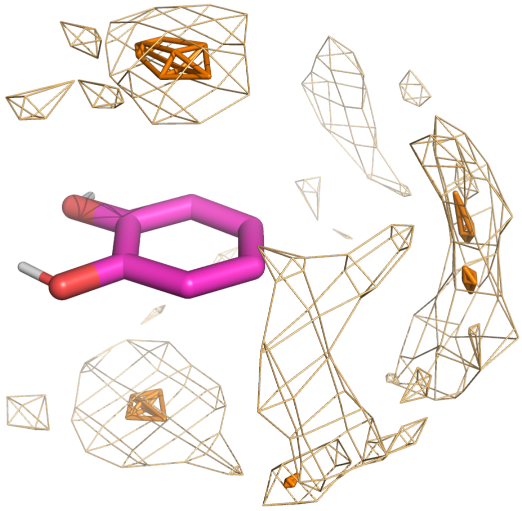

Figure S1. Interaction profile of aromatic rings of Phe, Tyr, and Trp, around catechol (purple). light and dark orange meshes indicate the profile with different thresholds.

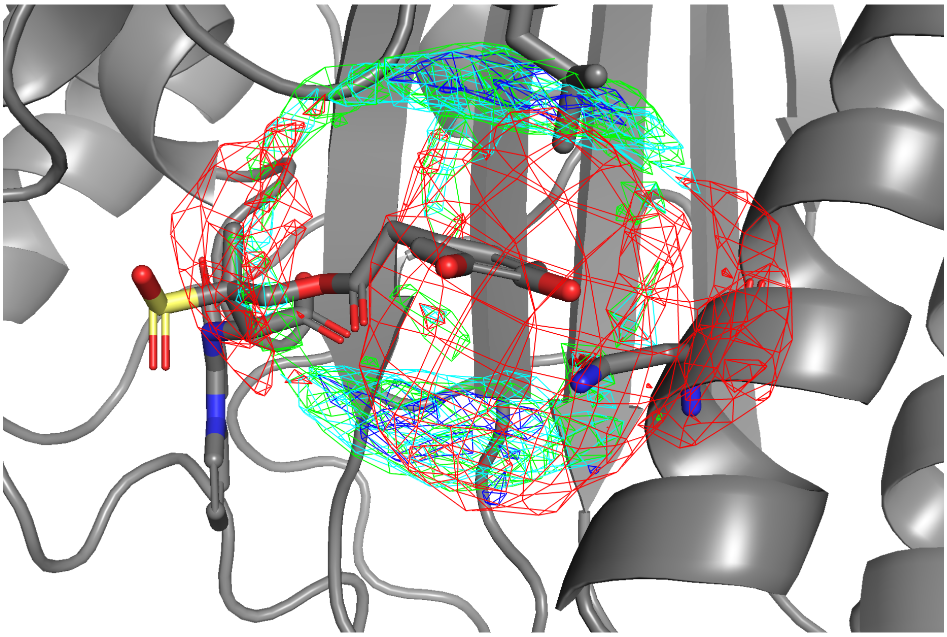

Figure S2. A superimposition of residue interaction profile of catechol to a β-Lactamase Inhibitor, LN-1-255 (PDBID: 3D4F). LN-1-255 has a catechol substituent and the profile was superimposed to it in accordance with the 3D structure of catechol. Green, cyan, gray, blue, and red meshes indicate profiles of hydrophobic, hydrophilic, aromatic, basic, and acidic residues, respectively.
